# Sepsis‐Induced Delirium Progresses Tau Pathology in P301S Mouse Model of Alzheimer's Disease

**DOI:** 10.1002/alz70855_107655

**Published:** 2025-12-24

**Authors:** Camryn Smith, Fiona E. Harrison

**Affiliations:** ^1^ Vanderbilt University, Nashville, TN, USA; ^2^ Department of Medicine, Vanderbilt University Medical Center, Nashville, TN, USA; ^3^ Vanderbilt Brain Institute, Vanderbilt University, Nashville, TN, USA; ^4^ Vanderbilt University Medical Center, Nashville, TN, USA; ^5^ Vanderbilt Memory & Alzheimer's Center, Vanderbilt University Medical Center, Nashville, TN, USA

## Abstract

**Background:**

Delirium is a severe complication of sepsis characterized by acute cognitive dysfunction. Alzheimer's disease (AD) is a neurodegenerative disorder marked by chronic cognitive decline and accumulation of phosphorylated tau protein. Recent evidence suggests a potential link between sepsis‐induced delirium and the progression of AD pathology, particularly tau pathology. This study investigates the impact of sepsis‐induced delirium on tau pathology using a long‐term model of sepsis in P301S mice, which express mutant human tau protein.

**Method:**

20‐week‐old wild‐type C57BL/6 mice and transgenic P301S mice were divided into four groups: WT saline, WT cecal slurry (CS), P301S saline, and P301S CS. Sepsis was induced via intraperitoneal injection of cecal slurry. Behavioral assessments conducted between day 3 post‐injection and day 8 included Y‐maze, nest building, rotarod, tail suspension, elevated zero maze, open‐field, novel object recognition, and water maze tests. Electroencephalography (EEG) performed post‐surgery, during sickness time, and throughout the behavioral testing week to evaluate brain activity and characterize a delirium‐like state. Postmortem biochemical analyses performed post‐SAC on day 8 encompassed fluorescent immunohistochemistry for microglia and phosphorylated tau, enzyme‐linked immunosorbent assay for phosphorylated tau, and multiplex assays for inflammatory markers.

**Result:**

Preliminary data indicated CS‐treated mice exhibited increased depressive‐like behavior in the tail suspension test compared to saline controls at TIMINGS in the absence of other sepsis induced cognitive deficits. Immunohistochemistry revealed significant phosphorylated tau accumulation and microglial activation in P301S groups, particularly in CS‐treated mice. EEG data reveal clear differences between sick and recovered mice.

**Conclusion:**

The preliminary findings suggest that sepsis‐induced delirium may contribute to the progression of tau pathology in P301S mice. While cognitive deficits were not apparent in initial behavioral tests, the observed increase in phosphorylated tau and microglial activation in P301S CS mice indicates a potential long‐term impact of sepsis on AD‐like pathology. Further data collection and analysis are necessary to fully elucidate the relationship between sepsis‐induced delirium and tau pathology progression in this model.

